# Multi-Omics Integration and Causal Inference Identify HSD17B1 as a Potential Nobiletin Target Linking Neurosteroid Metabolism to Alzheimer’s Disease

**DOI:** 10.3390/ijms27114756

**Published:** 2026-05-25

**Authors:** Renjie Gao, Chenqu Lyu, Yumeng Gu, Ruixiao Hao, Chao Wang, Xin Li

**Affiliations:** 1 Department of Geriatrics, The Second Hospital of Tianjin Medical University, Tianjin 300211, China; 2 The First Clinical Medical College, Anhui Medical University, Hefei 231241, China; lvchenqu@163.com; 3 Department of Neurology, The Second Hospital of Tianjin Medical University, Tianjin 300211, China; 4 Tianjin Interdisciplinary Innovation Centre for Health and Meteorology, Tianjin 300211, China

**Keywords:** Alzheimer’s disease, nobiletin, HSD17B1, network pharmacology, WGCNA, ensemble machine learning, SHAP, Mendelian randomization, immune infiltration, single-cell RNA-seq, spatial transcriptomics, molecular docking, molecular dynamics

## Abstract

Alzheimer’s disease (AD) is characterized not only by neuronal dysfunction but also by profound remodeling of the brain microenvironment, including immune–glial activation and metabolic dysregulation. Increasing evidence also implicates neurosteroid-related pathways in AD and dementia. Nobiletin has shown neuroprotective effects in AD-related models, but its upstream human targets and mechanism-based translational relevance remain insufficiently defined. Here, we integrated multi-omics analyses, interpretable machine learning, causal inference, structural modeling, and experimental validation to identify candidate nobiletin-associated molecular nodes in AD. HSD17B1 consistently emerged as a central AD-associated candidate across multiple analytical layers and showed reproducible discriminatory performance in independent validation cohorts. SHAP analysis further identified HSD17B1 as a major contributor to the optimal predictive model, while Mendelian randomization supported a protective association between genetically increased HSD17B1 expression and AD risk. Immune infiltration, single-cell, and spatial transcriptomic analyses linked HSD17B1 to glia-associated remodeling and regionally heterogeneous expression patterns in AD. Molecular docking and molecular dynamics simulations supported the structural feasibility of nobiletin binding to HSD17B1, and in an Aβ1–42-induced SH-SY5Y cell model, nobiletin increased HSD17B1 expression at both the mRNA and protein levels. Together, these findings support HSD17B1 as an AD-associated and nobiletin-responsive candidate molecular node, highlight a potential connection between nobiletin and neurosteroid-related regulation, and provide an integrated framework for target prioritization and validation in AD.

## 1. Introduction

Alzheimer’s disease (AD) is the most common cause of dementia and a growing global health challenge in rapidly ageing societies [1,2,3]. Although anti-amyloid-β monoclonal antibodies such as lecanemab and donanemab have shown statistically significant slowing of cognitive and functional decline in early AD, their clinical use remains limited by safety concerns, amyloid-related imaging abnormalities, cost, infusion burden, and the need for biomarker confirmation [4,5,6,7,8,9]. These limitations highlight the need for complementary strategies that target broader mechanisms of AD, including neuroinflammation, synaptic dysfunction, vascular injury, oxidative stress, and metabolic dysregulation [2,10,11].

Brain ageing and dementia risk are shaped not only by genetic susceptibility but also by long-term interactions among diet, lifestyle, environmental exposure, metabolic status, work-related stress, sleep and activity patterns, and other behavioral factors [12]. Across different populations and cultural settings, dietary patterns rich in whole foods, fruits, vegetables, and naturally occurring antioxidant compounds have been associated with healthier cognitive ageing, whereas adverse exposures such as air pollution, endocrine-disrupting chemicals, metabolic stress, and unhealthy behavioral patterns may contribute to oxidative damage, inflammatory activation, and accelerated age-related decline [12,13,14]. In this context, diet-derived bioactive molecules and antioxidant-related nutrients, including vitamin C, have attracted increasing interest as accessible but complementary modulators of ageing-related biological pathways [15,16,17]. Rather than replacing disease-modifying therapies, such compounds may provide mechanistic clues for understanding how antioxidant, anti-inflammatory, metabolic, and neuroendocrine pathways intersect in AD [18].

**Figure 1:**
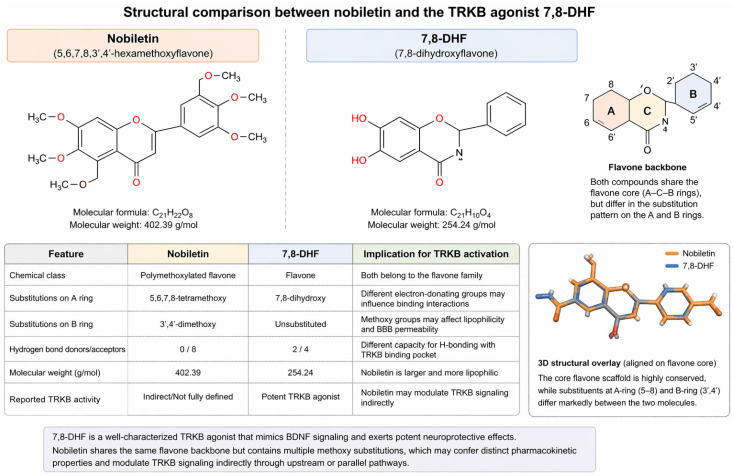
Structural comparison between nobiletin and the TRKB agonist 7,8-DHF.

Nobiletin, a polymethoxylated flavone enriched in citrus peel, is one such diet-derived compound. It has attracted attention for its neuroprotective, anti-inflammatory, antioxidant, and metabolism-modulating properties [19]. Preclinical studies suggest that nobiletin can improve cognitive and synaptic phenotypes and attenuate inflammatory signaling in AD-related models [20,21,22,23]. Structurally, nobiletin shares the conserved flavone backbone with several neuroactive flavones, including 7,8-dihydroxyflavone (7,8-DHF), a selective TRKB agonist with potent neurotrophic activity [24]. However, unlike 7,8-DHF, which contains 7,8-dihydroxy substitutions and directly activates BDNF/TRKB-related signaling, nobiletin contains multiple methoxy groups, suggesting distinct physicochemical properties and potentially different target-engagement mechanisms (Figure 1). Related flavones and plant-derived natural compounds have been reported to act through anti-inflammatory and antioxidant pathways, mitochondrial regulation, and metabolic remodeling [16,17,18,19]. Nevertheless, the upstream targets and target-centered mechanisms of nobiletin in AD remain insufficiently defined.

Emerging evidence implicates neurosteroid metabolism, estrogen-related signaling, and sex hormone balance in brain resilience, neuroinflammation, synaptic regulation, and cognitive ageing [25,26,27]. These pathways are particularly relevant to AD because sex-specific differences in disease risk and progression may partly reflect changes in local steroid metabolism and estrogenic signaling during ageing. Given the multi-target properties of flavonoids and their potential interactions with steroid-related pathways, nobiletin may influence AD biology through neurosteroid-associated regulatory nodes. HSD17B1 is especially relevant in this context because it catalyzes the conversion of estrone to estradiol and may thereby affect local estrogenic tone, inflammatory balance, and microenvironmental homeostasis [26,27].

In this study, we integrated network pharmacology, bulk transcriptomics, WGCNA, interpretable machine learning, Mendelian randomization, immune infiltration analyses, single-cell RNA sequencing, spatial transcriptomics, molecular docking, molecular dynamics simulation, and cell-based validation to identify candidate nobiletin-associated molecular nodes in AD [28,29,30,31]. Through this multi-layered framework, we prioritized HSD17B1 as an AD-associated and nobiletin-responsive candidate target, linking nobiletin to neurosteroid-related regulation and providing a mechanism-oriented basis for further investigation.

## 2. Results and Discussion

### 2.1. Target Identification and Differential Expression Analyses of Nobiletin in AD

**Figure 2:**
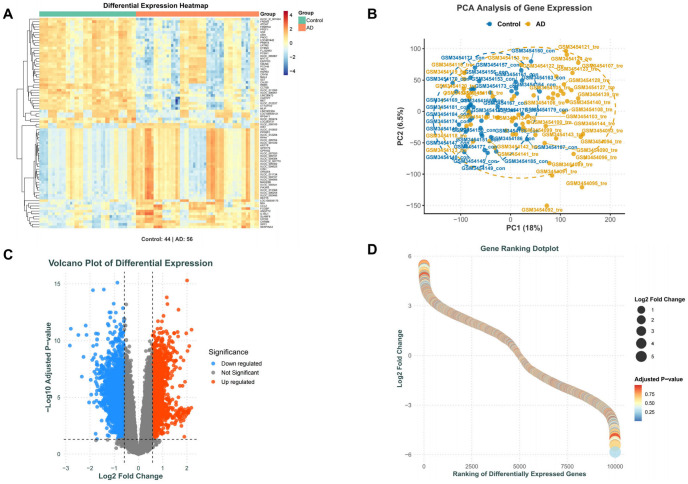
Differential expression analyses in the training cohort (GSE122063). (**A**) Heatmap clustering of AD (n = 56) and controls (n = 44); (**B**) PCA separation; (**C**) differentially expressed genes (DEGs), with dashed lines indicating the thresholds of adjusted *p* < 0.05 and |log2FC| > 0.58; (**D**) ranked DEGs.

A total of 327 putative nobiletin-related targets were compiled after integrating predictions from PharmMapper, CTD, and SwissTargetPrediction and removing duplicate entries. To identify AD-related transcriptional alterations relevant to these candidate targets, differential expression analyses were performed in the training cohort (GSE122063). As shown in Figure 2A, unsupervised clustering clearly separated AD samples from controls, indicating substantial transcriptomic differences between the two groups. Principal component analysis further supported this separation, with AD and control samples occupying distinct regions in the projection space (Figure 2B). A total of 3410 differentially expressed genes (DEGs) were identified, including 1535 upregulated and 1875 downregulated genes (Figure 2C). The ranked DEG distribution is shown in Figure 2D.

These results indicate that the training cohort captured a robust AD-associated transcriptional signature and provided a suitable basis for subsequent target prioritization. The clear group separation observed in both clustering and PCA analyses supports the biological relevance of the downstream integrative analyses.

### 2.2. WGCNA and Identification of Candidate Nobiletin-Related Genes

**Figure 3:**
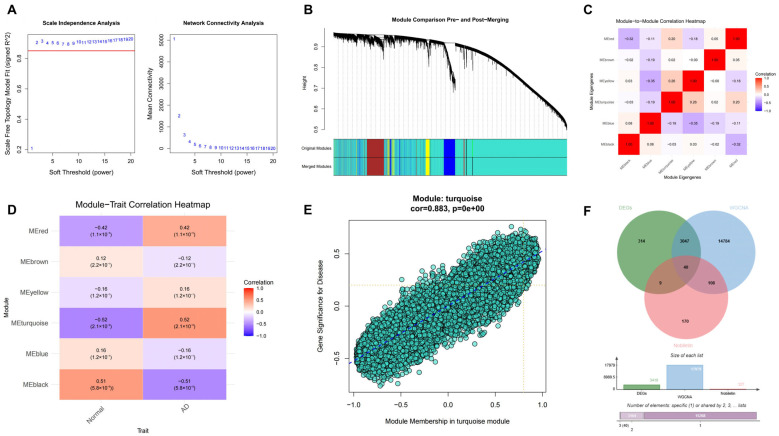
Co-expression analyses identify AD-associated modules (WGCNA; GSE122063). (**A**) Soft-threshold selection (β = 2); (**B**) Module assignment; (**C**) Module relationships; (**D**) Turquoise module associated with AD (cor = 0.52, *p* = 2.1 × 10^−8^); (**E**) Module consistency; (**F**) Overlap genes.

To further define disease-relevant gene modules, weighted gene co-expression network analyses (WGCNA) were performed on the training dataset. The optimal soft-thresholding power was determined to be 2 (Figure 3A), and dynamic tree cutting identified six co-expression modules (Figure 3B). The relationships between these modules were visualized in a module correlation heatmap (Figure 3C), which shows the pairwise correlations between different gene modules. Among these, the turquoise module showed the strongest positive association with AD status (cor = 0.52, *p* = 2.1 × 10^−8^; Figure 3D). In addition, genes within this module displayed strong concordance between module membership and eigengene-based connectivity (r = 0.883, *p* < 0.001; Figure 3E), indicating high internal consistency. Intersecting DEGs, turquoise-module genes, and nobiletin-related targets yielded 40 overlapping genes (Figure 3F).

These analyses reduced the candidate space from a broad set of predicted targets to a more focused group of genes supported by both AD-associated differential expression and coordinated co-expression structure. The strong disease correlation of the turquoise module suggests that these overlap genes are more likely to reflect biologically relevant processes than isolated transcript changes alone.

### 2.3. PPI Network Construction and Core Gene Screening

**Figure 4:**
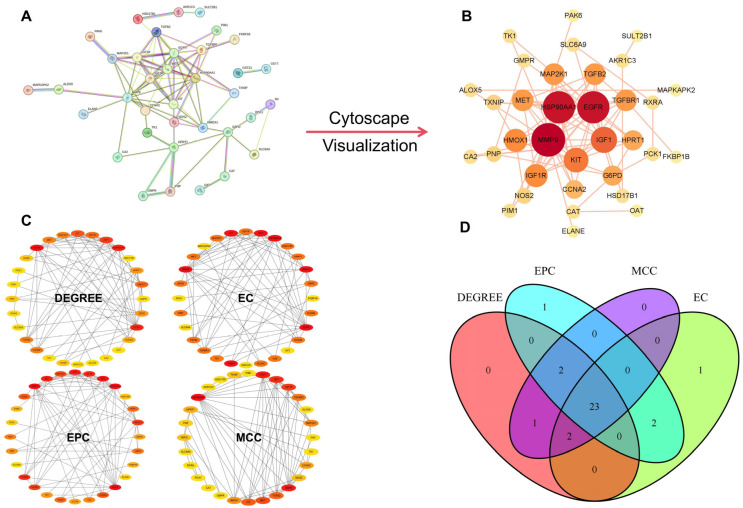
PPI network and core gene screening. (**A**) STRING PPI network (Homo sapiens; confidence = 0.400); (**B**) Cytoscape view by degree; (**C**) Top genes ranked by multiple methods; (**D**) Shared core genes.

To evaluate functional relationships among the overlap genes, a protein–protein interaction (PPI) network was constructed using STRING v12.5. After removal of unconnected nodes, the network contained 36 nodes and 81 edges (Figure 4A). Cytoscapev3.9.1 visualization of the PPI network (Figure 4B) shows the interaction structure, with hub genes highlighted by their degree of connectivity. Hub prioritization using four cytoHubba algorithms (DEGREE, EC, EPC, and MCC) identified 28 highly ranked genes (Figure 4C). Integration of these rankings yielded 23 shared core genes (Figure 4D).

The PPI analyses provided an additional layer of prioritization by focusing on genes occupying central positions in the inferred interaction network. Because highly connected nodes may participate in coordinated disease-relevant processes, these 23 genes were considered strong candidates for subsequent diagnostic and mechanistic evaluation.

### 2.4. Machine Learning Prioritization Identifies HSD17B1 as a Core AD-Associated Gene

**Figure 5:**
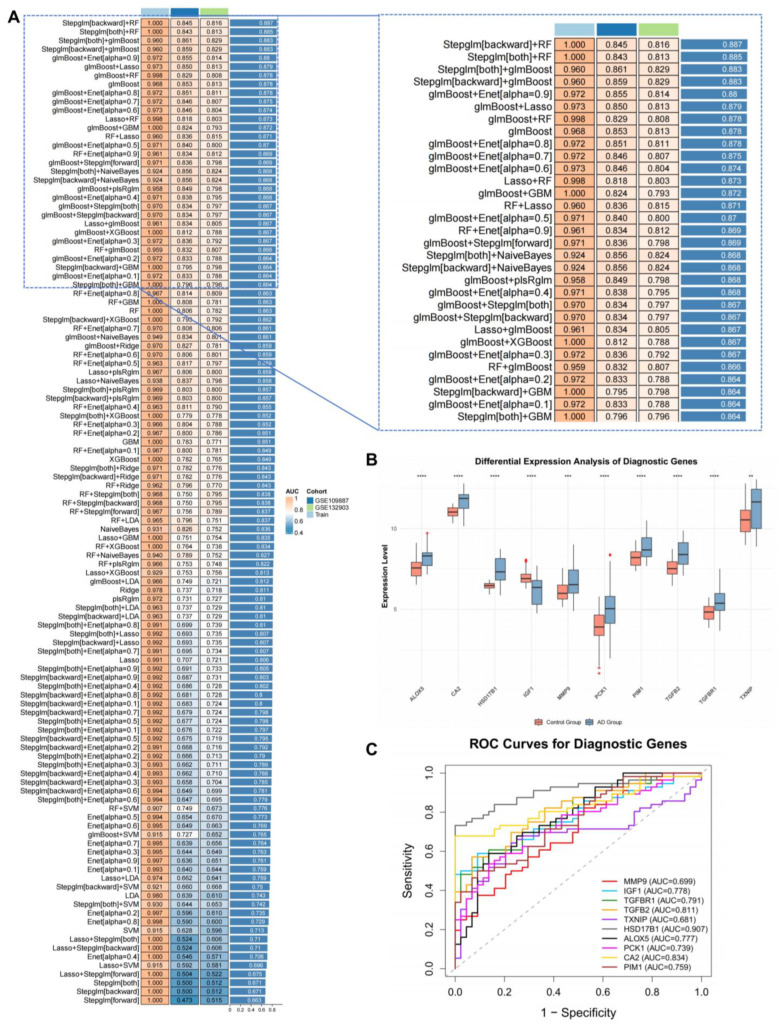
Machine-learning selection of diagnostic genes. (**A**) Model comparison (best: Stepglm[backward]+RF; validation AUC = 0.887); (**B**) Expression differences in selected genes (GSE122063); (**C**) ROC performance of key genes (HSD17B1 highest; AUC = 0.907). ** *p* < 0.01, *** *p* < 0.001 and **** *p* < 0.0001.

The diagnostic value of the candidate genes was next evaluated using 113 machine-learning model combinations, consistent with the growing use of omics-based and interpretable machine-learning strategies for AD biomarker discovery [32,33]. Among the tested approaches, the Stepglm[backward] + RF model showed the best performance in the validation cohort, achieving an AUC of 0.887 (Figure 5A). The genes retained by the optimal model exhibited clear expression differences between AD and control samples (Figure 5B). ROC analyses further demonstrated strong discriminatory performance for individual genes, among which HSD17B1 showed the highest AUC (0.907; Figure 5C).

These results indicate that HSD17B1 was not only retained through multi-step bioinformatic screening but also emerged as the strongest single-gene discriminator in the optimized diagnostic framework. The consistency of this prioritization across target prediction, transcriptomic filtering, network analyses, and supervised modeling supports HSD17B1 as a biologically meaningful AD-associated candidate rather than a dataset-specific artifact.

### 2.5. SHAP-Based Model Interpretation Supports the Contribution of HSD17B1

**Figure 6:**
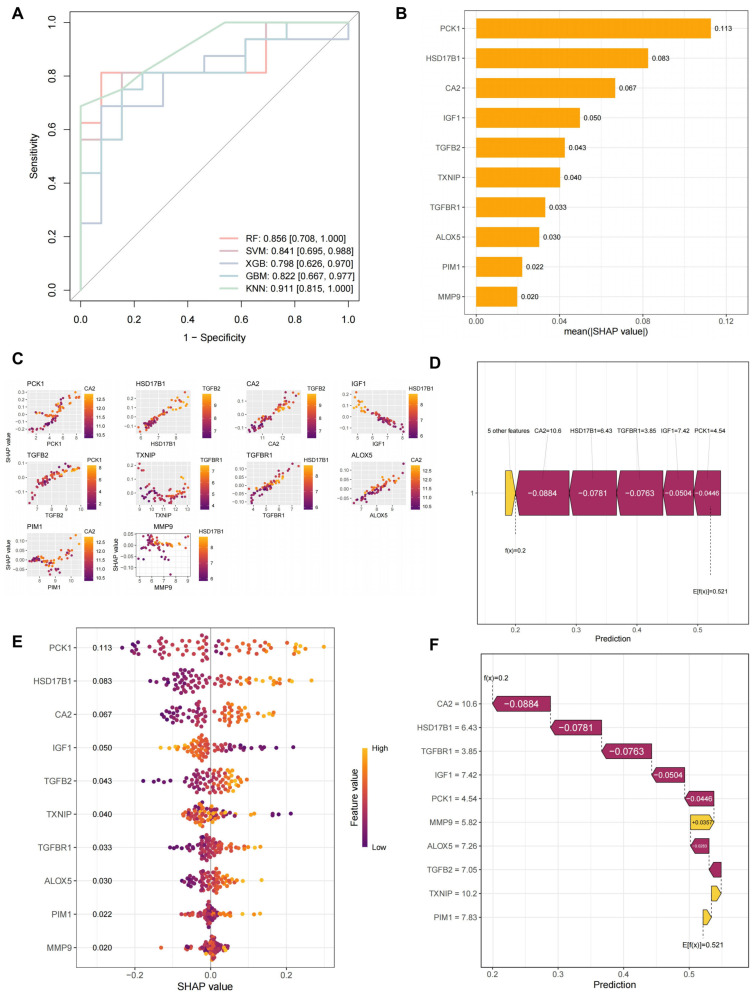
Explainable model interpretation (SHAP; GSE122063). (**A**) Classifier ROC comparison (KNN best; AUC = 0.911); (**B**) Top features by SHAP; (**C**) Gene–gene correlations; (**D**) Marginal-effect curves; (**E**) SHAP vs. expression; (**F**) Single-sample explanation.

To further assess model robustness and interpret feature contributions, an explainable machine-learning framework was applied, as explainable artificial intelligence has become increasingly important for improving the interpretability of AD classification models [34]. Among several classifiers, the KNN model achieved the best performance, with an AUC of 0.911 (95% CI: 0.815–1.000; Figure 6A). SHAP analyses ranked PCK1 as the top contributor, followed by HSD17B1, CA2, and IGF1 (Figure 6B). Correlation analyses showed coordinated expression relationships among several diagnostic genes, including PCK1–CA2, HSD17B1–TGFB2, and CA2–TGFBR1 (all r > 0.5; Figure 6C). ICE analyses indicated that CA2, HSD17B1, TGFBR1, and IGF1 exerted notable marginal effects on model output (Figure 6D). In addition, the SHAP-expression relationship showed that higher HSD17B1 expression was associated with larger positive SHAP values, supporting a stable contribution of HSD17B1 to classification toward the disease state (Figure 6E). The force plot further illustrated that HSD17B1 acted together with other features to shift the predicted probability toward AD (Figure 6F).

These findings strengthen the interpretation that HSD17B1 is not merely statistically associated with AD but also contributes meaningfully to model decisions within a multigene predictive context. Although HSD17B1 was not the single highest SHAP-ranked feature, its consistent contribution across multiple interpretability analyses supports its importance in the optimized diagnostic signature.

### 2.6. Functional Enrichment Suggests a Link Between HSD17B1 and Steroid-Related Pathways

**Figure 7:**
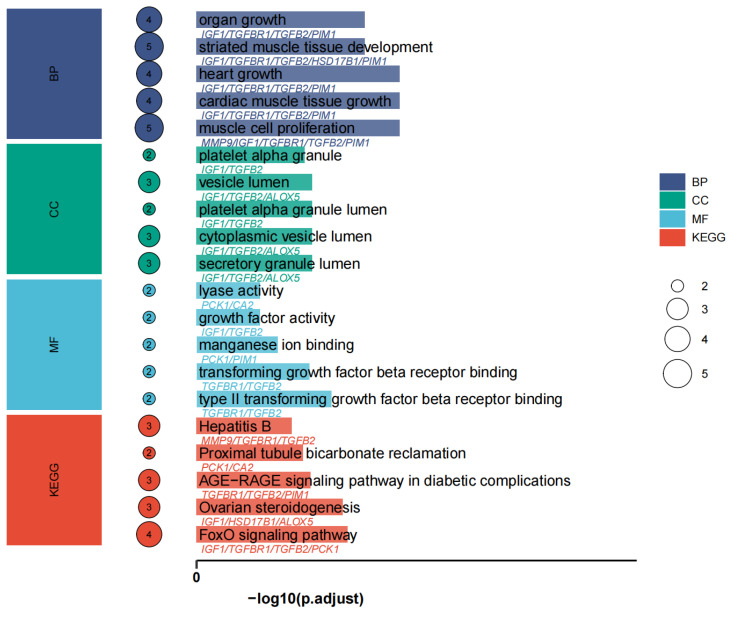
Functional enrichment of diagnostic genes. GO enrichment (adjusted *p* < 0.05); KEGG enrichment (adjusted *p* < 0.05), including ovarian steroidogenesis.

To explore the biological functions of the prioritized diagnostic genes, GO and KEGG enrichment analyses were performed. GO terms were enriched in processes related to growth, striated muscle tissue development, heart growth, and muscle cell proliferation, while cellular component terms were largely associated with vesicle- and granule-related compartments (Figure 7). Molecular function enrichment highlighted lyase activity, growth factor activity, manganese ion binding, and transforming growth factor β receptor binding. KEGG analyses identified several enriched pathways, including ovarian steroidogenesis, in which HSD17B1 was implicated together with genes such as IGF1 and ALOX5.

Although some enriched GO terms were not directly brain-specific, the KEGG signal linking HSD17B1 to steroidogenesis is notable in the context of AD. HSD17B1 encodes 17β-hydroxysteroid dehydrogenase type 1, an enzyme involved in estrone-to-estradiol conversion, and this finding is therefore consistent with growing evidence that estrogen-related and neurosteroid-associated pathways may influence synaptic function, neuroinflammation, and brain ageing [25,26,35,36,37,38]. Rather than establishing a definitive mechanism, these enrichment results support steroid-metabolic regulation as a plausible biological context for interpreting the role of HSD17B1 in AD. The broader implications of HSD17B1 within steroidogenic networks, androgen–estrogen balance, and sex differences in AD are discussed below.

### 2.7. Mendelian Randomization Supports a Protective Association of HSD17B1 with AD

**Figure 8:**
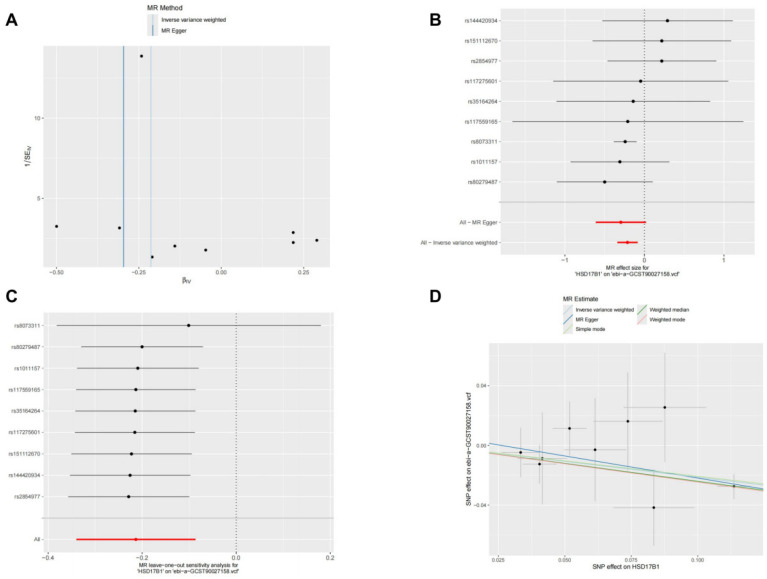
Mendelian randomization supports HSD17B1 as a protective factor for AD. (**A**) Overall effects (IVW and MR-Egger); (**B**) SNP-level estimates; (**C**) Leave-one-out sensitivity; (**D**) Consistency across methods (cis-eQTL: eQTLGen; outcome: AD GWAS ebi-a-GCST90027158).

To evaluate causal relevance, Mendelian randomization (MR) analyses were performed for ten candidate genes. Among them, only HSD17B1 showed a positive result. The IVW and MR-Egger analyses both indicated a negative effect estimate, suggesting that genetically elevated HSD17B1 expression was associated with reduced AD risk (Figure 8A). SNP-level analyses showed that all nine independent HSD17B1-related variants yielded negative causal estimates (Figure 8B). Leave-one-out analysis demonstrated that no single variant disproportionately drove the overall result (Figure 8C). Moreover, multiple MR methods, including IVW, MR-Egger, weighted median, weighted mode, and simple mode, showed the same overall trend (Figure 8D).

This genetic evidence provides an important line of support beyond transcriptomic association. Because MR can reduce confounding and reverse causation, the consistent protective estimates observed here strengthen the prioritization of HSD17B1 as a candidate AD-relevant target [39,40]. At the same time, this interpretation should remain cautious, because the instruments were derived from whole-blood cis-eQTL data rather than brain-specific regulatory datasets.

### 2.8. External Validation Confirms the Diagnostic Value of HSD17B1

**Figure 9:**
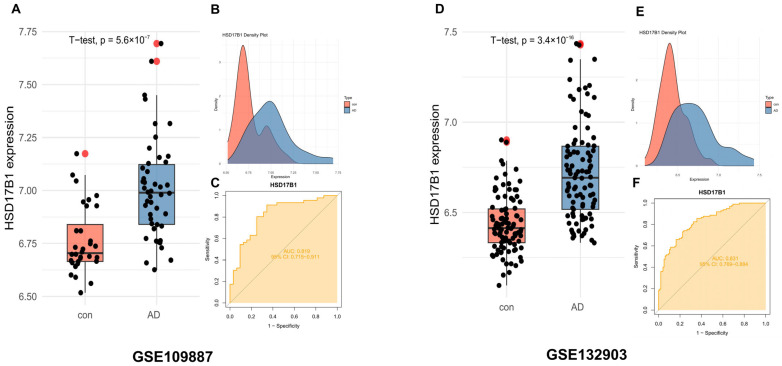
External validation of HSD17B1 in independent cohorts. (**A**–**C**) GSE109887: expression, density, and ROC; (**D**–**F**) GSE132903: expression, density, and ROC.

The diagnostic value of HSD17B1 was further assessed in two independent validation cohorts. In GSE109887, HSD17B1 expression was significantly higher in AD samples than in controls (*p* = 5.6 × 10^−7^), and ROC analysis yielded an AUC of 0.816 (95% CI: 0.715–0.911; Figure 9A–C). In GSE132903, HSD17B1 expression was again significantly elevated in AD (*p* = 3.4 × 10^−16^), with an AUC of 0.831 (95% CI: 0.768–0.884; Figure 9D–F).

The replication of both differential expression and diagnostic performance across two independent datasets substantially improves confidence in HSD17B1 as a reproducible marker. Although the AUC values are not sufficient to claim standalone clinical utility, their stability across cohorts supports the robustness of HSD17B1 as a prioritized AD-associated gene.

### 2.9. Immune Infiltration Analyses Reveal an Immunometabolic Association of HSD17B1

**Figure 10:**
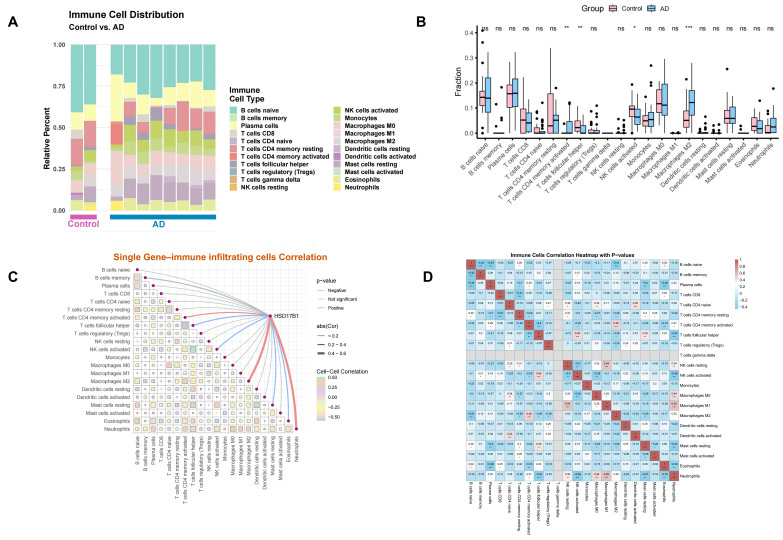
Immune infiltration analyses and association with HSD17B1. (**A**) Immune-cell composition; (**B**) Group comparison; (**C**) HSD17B1–immune correlations; (**D**) Immune correlation heatmap. *, ** and *** indicate *p* < 0.05, *p* < 0.01 and *p* < 0.001, respectively; ns indicates no statistical significance.

Immune infiltration analyses showed clear differences in immune-cell composition between AD and control samples. In AD, naive B cells, memory B cells, plasma cells, and CD8^+^ T cells were reduced, whereas activated NK cells, monocytes, and M2 macrophages were increased (Figure 10A,B). Correlation analyses centered on HSD17B1 revealed significant positive associations with multiple B-cell-related populations, including naive B cells, memory B cells, and plasma cells, together with negative associations with some activated immune cell populations (Figure 10C). The immune correlation heatmap further suggested coordinated relationships between adaptive and innate immune compartments (Figure 10D).

These results point to a potential immunometabolic dimension of HSD17B1 in AD. Neuroinflammation is increasingly recognized as an integral component of AD progression, including contributions from peripheral and innate immune compartments [41,42]. In addition, steroid-related pathways are known to influence inflammatory and metabolic programs in a cell-context-dependent manner [43]. In this setting, the association between HSD17B1 and B-cell-related infiltration patterns raises the possibility that HSD17B1 may contribute to immune homeostasis or immune remodeling in AD. However, these findings remain correlative and require mechanistic validation.

### 2.10. Single-Cell RNA-Seq Analyses Reveal Cell-Type Specificity of HSD17B1 in AD

**Figure 11:**
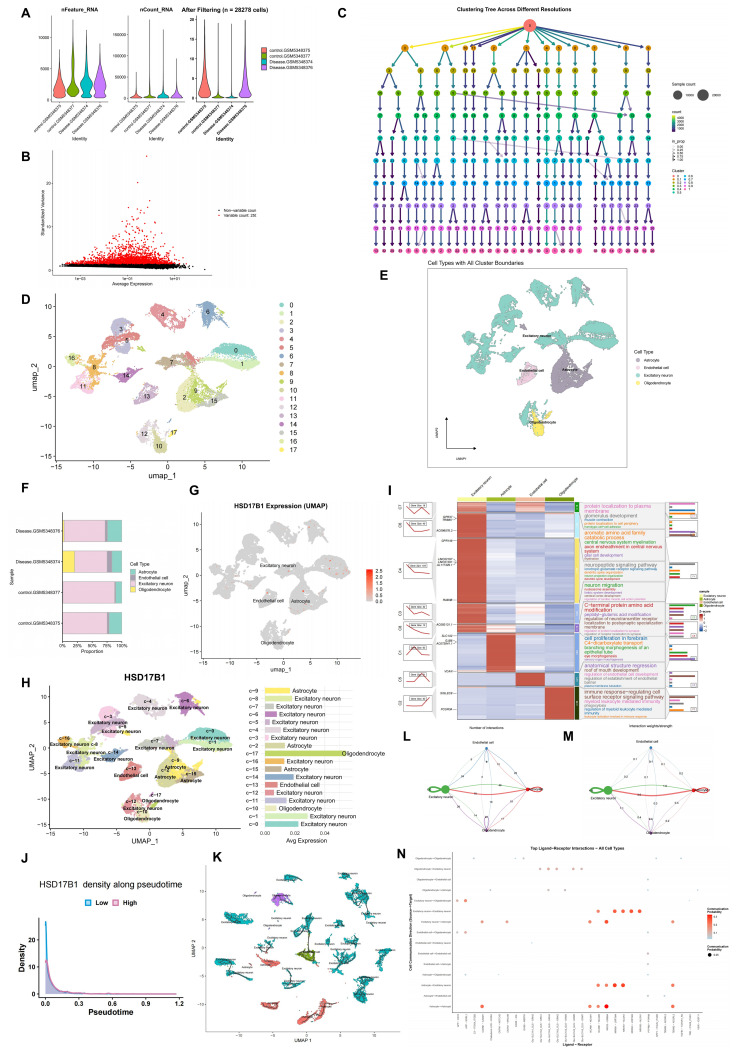
Single-cell RNA-seq analyses show cell-type specificity of HSD17B1 (GSE175814). (**A**) QC summary; (**B**) Variable genes; (**C**,**D**) Clustering; (**E**,**F**) Cell-type annotation and proportions; (**G**,**H**) HSD17B1 expression; (**I**–**K**) Trajectory/pseudotime patterns; (**L**–**N**) Cell–cell communication.

After quality control, 28,278 high-quality cells from four samples were retained for single-cell analyses. Quality metrics were comparable across samples (Figure 11A), and 2500 highly variable genes were selected (Figure 11B). Clustering at resolution 0.3 identified 18 transcriptionally distinct clusters (Figure 11C,D). Cell-type annotation showed that excitatory neurons were the dominant population, followed by astrocytes, endothelial cells, and oligodendrocytes (Figure 11E,F). Disease samples showed a relative reduction in neuronal populations and an increase in glial populations, consistent with AD-associated cellular remodeling. HSD17B1 displayed clear cell-type specificity, with enriched expression in astrocyte clusters and lower expression in subsets of neurons and endothelial cells (Figure 11G,H). Functional enrichment of cluster markers revealed distinct biological programs across cell types (Figure 11I), and pseudotime analysis suggested dynamic changes in HSD17B1 expression along inferred trajectories (Figure 11J,K). CellChat analysis indicated strong neuron–neuron communication and prominent astrocyte–neuron interactions, whereas endothelial participation was comparatively limited (Figure 11L–N).

These findings suggest that HSD17B1 is preferentially linked to astrocyte-associated transcriptional programs in AD. Given the growing recognition that astrocytes undergo reactive-state transitions and metabolic reprogramming during disease progression [44,45], the enrichment of HSD17B1 in astrocytes provides an important cell-type context for interpreting its potential role in the AD microenvironment.

### 2.11. Spatial Transcriptomics Analyses Reveal Region-Specific HSD17B1 Expression Patterns

**Figure 12:**
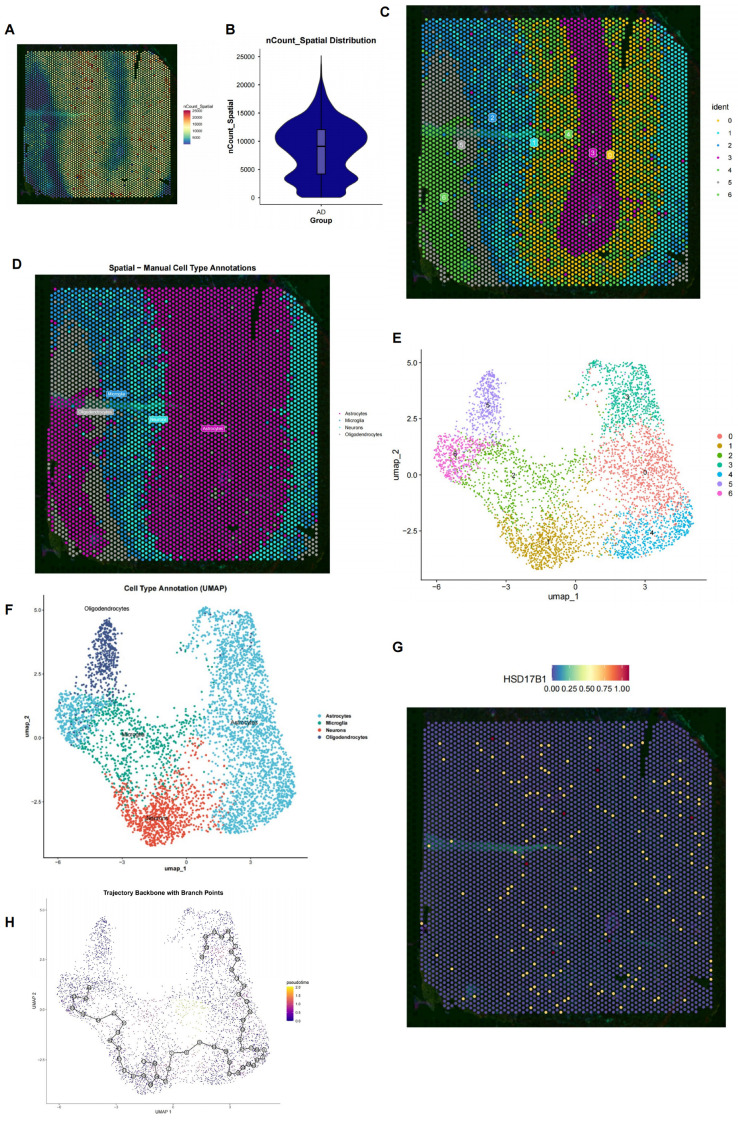
Spatial transcriptomics analyses reveal regional heterogeneity of HSD17B1 (GSE220442; 10× Visium). (**A**–**C**) Spatial clusters/regions; (**D**–**F**) Cell-type distributions; (**G**) Spatial map of HSD17B1; (**H**) Spatial trajectory-related patterns.

Spatial transcriptomic profiling, which enables transcriptome-scale mapping of gene expression within tissue architecture [46,47], revealed marked regional heterogeneity in transcript abundance and cluster distribution across the tissue section (Figure 12A–F). Deconvolution analysis suggested spatially organized distributions of astrocytes, microglia, neurons, and oligodendrocytes. HSD17B1 expression was heterogeneous, with discrete high-expression foci superimposed on broader low-expression regions (Figure 12G). Integration of spatial position with clustering and trajectory inference suggested that late-pseudotime transcriptional programs were enriched in regions with higher transcriptional activity and stronger glial signatures (Figure 12H).

These results indicate that HSD17B1 is not uniformly expressed across AD brain tissue but instead follows a regionally structured pattern. Together with the single-cell results, this supports the idea that HSD17B1 is embedded within localized, cell-type-dependent microenvironmental remodeling rather than reflecting a generalized bulk-tissue signal.

### 2.12. Structural Modeling Supports the Feasibility of Nobiletin–HSD17B1 Interaction

**Figure 13:**
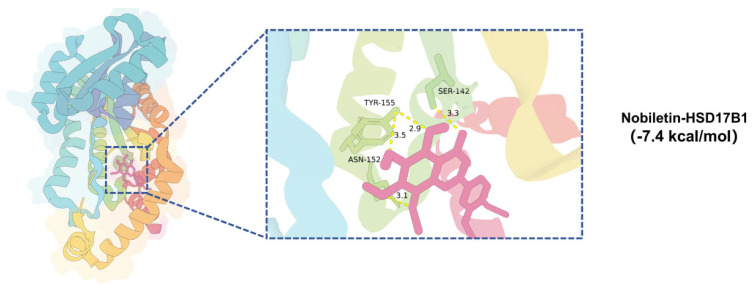
Molecular docking of nobiletin with HSD17B1. Docking pose in HSD17B1 (PDB: 6MNE) with key contacts (SER-142, ASN-152, TYR-155); binding energy = −7.4 kcal/mol.

**Figure 14:**
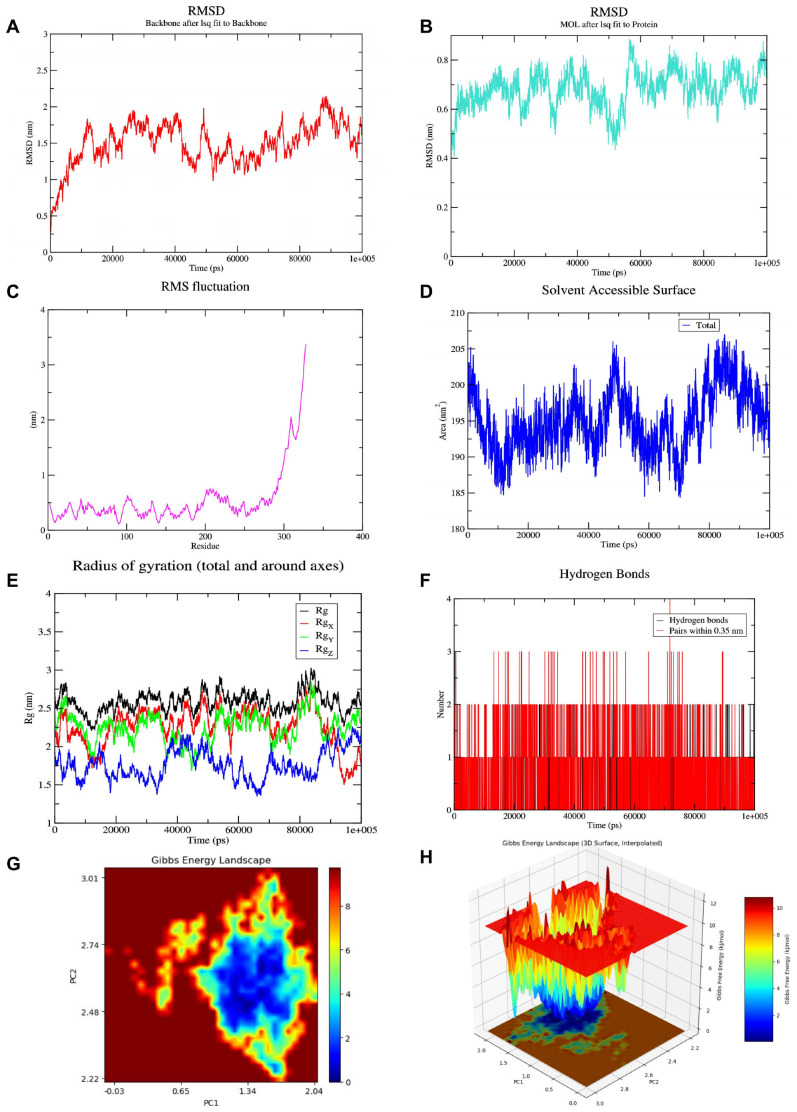
Molecular dynamics simulation of the nobiletin–HSD17B1 complex (100 ns). (**A**) Protein RMSD; (**B**) Ligand RMSD; (**C**) RMSF; (**D**) SASA; (**E**) Rg; (**F**) Hydrogen bonds; (**G**,**H**) Free-energy landscapes.

To evaluate whether HSD17B1 could plausibly function as a nobiletin-responsive molecular node, molecular docking and molecular dynamics (MD) simulations were performed. Docking analysis showed that nobiletin formed a stable complex with HSD17B1, with a binding energy of −7.4 kcal/mol and hydrogen-bond interactions involving SER-142, ASN-152, and TYR-155 (Figure 13). In the 100 ns MD simulation, the protein backbone RMSD stabilized after the early stage of the trajectory, while ligand RMSD remained relatively low, indicating persistent binding within the pocket (Figure 14A,B). RMSF analysis showed limited residue fluctuation in most regions, and SASA and radius of gyration remained relatively stable throughout the simulation (Figure 14C–E). The complex also maintained 2–3 hydrogen bonds during the simulation (Figure 14F), and both two-dimensional and three-dimensional free-energy landscapes supported the presence of a preferred low-energy conformational state (Figure 14G,H).

Taken together, these structural analyses support the feasibility of a stable nobiletin–HSD17B1 interaction. Although docking and MD cannot by themselves establish direct biochemical modulation or in vivo target engagement, they provide a structural rationale for considering HSD17B1 a plausible molecular node through which nobiletin may exert part of its biological effects. This interpretation is consistent with the broader view that flavonoids may exert neuroprotective effects through multi-target mechanisms rather than a single canonical pathway [48,49].

### 2.13. Experimental Validation Confirms Nobiletin-Induced Upregulation of HSD17B1

**Figure 15:**
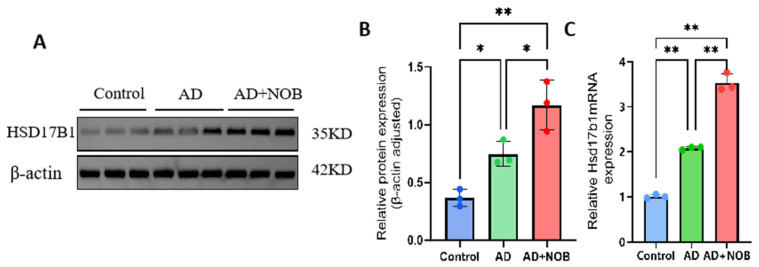
Nobiletin upregulates HSD17B1 in an AD-like cell model. (**A**) Western blot of HSD17B1 (β-actin control), (**B**) protein quantification, and (**C**) qRT-PCR of HSD17B1 mRNA in Control, AD, and AD+NOB groups. * *p* < 0.05, ** *p* < 0.01.

To experimentally test the relationship between nobiletin and HSD17B1, an Aβ_1–42_-induced AD-like SH-SY5Y cell model was used. Western blot analysis showed that HSD17B1 protein expression was higher in the AD group than in the control group and was further increased after nobiletin treatment (Figure 15A,B). Densitometric quantification confirmed that HSD17B1 protein levels were significantly increased in AD versus control (*p* < 0.05) and further increased in AD + NOB (nobiletin) versus AD (*p* < 0.01). qRT-PCR analysis showed the same overall pattern at the mRNA level, with significant upregulation in AD and additional elevation after nobiletin treatment (Figure 15C).The amplification and melt-curve plots for HSD17B1 and β-actin are provided in Figure S1, and the original full-length western blots for HSD17B1 and β-actin are provided in Figure S2.

These experiments provide direct support that HSD17B1 is responsive to nobiletin under AD-like conditions. Importantly, the cell-based validation complements the computational, transcriptomic, and genetic evidence presented above. These findings are also consistent with recent experimental studies showing that nobiletin can modulate neuroinflammatory and AD-like pathological responses in cellular and animal models [50,51]. At the same time, the findings do not imply that HSD17B1 fully mediates all effects of nobiletin; rather, they support HSD17B1 as one component of a broader protective molecular network.

### 2.14. Biological Implications of HSD17B1 in Neurosteroid Metabolism and Sex Differences in AD

The potential relevance of HSD17B1 in AD extends beyond its diagnostic performance or predicted interaction with nobiletin. Historically, the neuroendocrine metabolism of progesterone and related progestins was systematically discussed by Karavolas and Hodges, who emphasized that steroid metabolites can be actively generated in neural and neuroendocrine tissues and may influence neuronal activity [52]. This classical work provides an important conceptual basis for considering steroid-metabolic enzymes, including 3β-HSD and HSD17B family members, as biologically relevant to brain function and disease. A key biological question is why a steroid-metabolic enzyme should matter in a neurodegenerative disorder. Accumulating evidence indicates that sex steroids and neurosteroids are not only peripheral endocrine signals but also locally regulated modulators of neuronal survival, synaptic plasticity, mitochondrial function, neuroinflammation, and glial responses [25,26,35,36,37,53,54,55]. HSD17B1 should therefore be interpreted within a broader steroidogenic network rather than as an isolated estrogen-related enzyme. In this network, 3β-HSD acts upstream by converting Δ5-3β-hydroxysteroids, such as pregnenolone and dehydroepiandrosterone, into Δ4-ketosteroids, including progesterone and androstenedione, which can subsequently feed into androgen- and estrogen-related pathways. HSD17B enzymes act at later steps by regulating interconversions between less active and more active sex steroids, including estrone-to-estradiol conversion and androgen-related steroid interconversions [38,56]. Therefore, the relationship among 3β-HSD, androgens, estradiol, and HSD17B1 is biologically important because it reflects a coordinated steroid-metabolic network rather than a single linear pathway.

### 2.15. Limitations

Although this study prioritizes HSD17B1 as a candidate nobiletin-responsive molecular node in AD, several limitations exist. First, the study provides mainly associative and hypothesis-generating evidence of HSD17B1 upregulation, and loss-of-function or gain-of-function experiments are still needed to determine whether HSD17B1 causally mediates the protective effects of nobiletin. Second, the SH-SY5Y cell line used in this study does not fully represent key AD-related cell types, especially astrocytes. Future validation should therefore be conducted in primary astrocytes, induced pluripotent stem cell-derived neural systems, or neuron–astrocyte co-culture models. Third, due to the difficulty of obtaining brain-derived eQTL data, whole-blood eQTL data were used to infer the causal relationship between HSD17B1 and AD risk, which may limit the brain-specific interpretation of the MR results. Future studies should prioritize brain-derived or cell-type-specific eQTL resources to improve the accuracy and biological relevance of causal inference. Fourth, although the AUC values for HSD17B1 were statistically significant across validation cohorts, they remain below the level typically required for a standalone clinical diagnostic biomarker. Therefore, HSD17B1 should be interpreted primarily as a mechanistically relevant research target rather than as an immediately applicable diagnostic marker. Furthermore, although we expanded the biological interpretation of HSD17B1 in relation to neurosteroid metabolism and sex differences, the present study did not directly measure steroid metabolites or perform sex-stratified mechanistic experiments. Future studies should quantify estrone, estradiol, androgens, progesterone-derived neurosteroids, and related enzymatic activities in male and female AD models to determine whether the nobiletin–HSD17B1 axis has sex-dependent functional relevance. Finally, although docking and molecular dynamics simulations support the structural feasibility of nobiletin binding to HSD17B1, direct functional validation remains lacking. Future studies should incorporate enzyme activity assays, binding assays, and HSD17B1 perturbation experiments to determine whether nobiletin directly modulates HSD17B1 enzymatic activity or downstream steroid-metabolic flux.

These limitations provide important directions for future research. Further experimental validation will be necessary to evaluate whether HSD17B1 functions as a nobiletin-responsive target and to clarify its role in AD-related neurosteroid regulation.

## 3. Materials and Methods

### 3.1. Collection of Nobiletin Targets

We retrieved the chemical structure and Simplified Molecular Input Line Entry System (SMILES) of nobiletin (CID: 72344) from the PubChem database. Subsequently, potential targets of nobiletin were screened using the PharmMapper, CTD, and SwissTargetPrediction databases, with the species restricted to Homo sapiens. After merging the targets retrieved from the three databases and removing duplicates, a comprehensive library of nobiletin-related targets was obtained.

### 3.2. GEO Dataset Acquisition

Gene expression microarray datasets for Alzheimer’s disease (AD) were obtained from the GEO database. GSE122063, containing transcriptomic data from 56 AD patients and 44 normal controls, was used as the training cohort to identify AD-related expression changes. GSE109887 (97 AD patients and 98 controls) and GSE132903 (46 AD patients and 32 controls) were used as external validation cohorts.

### 3.3. Differential Expression Analyses

After extraction of gene expression profiles from the control and AD groups, background correction and quantile normalization were performed. Raw expression values were then log2-transformed. Differential expression analyses were conducted using the limma package (3.64.3) in R (4.5.1). Genes meeting the criteria of adjusted *p* < 0.05 and |log2FC| > 0.585 in GSE122063 were defined as differentially expressed genes (DEGs).

### 3.4. WGCNA

The WGCNA package (1.73) in R was used to identify gene co-expression modules associated with AD-related traits. Outlier samples were first assessed by hierarchical clustering. The soft-thresholding power β was determined using the pickSoftThreshold function with a scale-free topology fit of R^2^ ≥ 0.9. The adjacency matrix was transformed into a topological overlap matrix (TOM), and genes with similar expression patterns were grouped into modules by average-linkage hierarchical clustering based on TOM-derived dissimilarity. Correlations between gene modules and clinical traits were then calculated and visualized. Genes from the most relevant module were intersected with nobiletin-related targets and significant DEGs to obtain candidate genes.

### 3.5. PPI Network Analyses

The STRING database was used to construct the protein–protein interaction (PPI) network for the intersecting genes. The species was set to Homo sapiens and the confidence score was set to 0.400. The resulting network was visualized in Cytoscape 3.9.1, and hub genes were further identified using the cytoHubba (0.1) plugin based on four algorithms: DEGREE, EC, EPC, and MCC.

### 3.6. Screening of Core Genes by Machine Learning

A total of 113 algorithm combinations derived from 12 machine-learning methods were used to construct diagnostic models. The methods included Random Forest (RF), Elastic Net (Enet), Gradient Boosting Machine (GBM), glmBoost, Least Absolute Shrinkage and Selection Operator (Lasso), Linear Discriminant Analysis (LDA), Naive Bayes, Partial Least Squares Regression Generalized Linear Models (plsRglm), Ridge Regression, Stepwise Generalized Linear Models (Stepglm), Support Vector Machines (SVM), and XGBoost. Feature selection and model construction were performed in the internal training cohort (GSE122063), and model generalizability was assessed in the two external datasets (GSE109887 and GSE132903). The model with the highest mean area under the curve (AUC) across training and validation cohorts was selected as the optimal model and used for hub-gene prioritization.

### 3.7. SHAP-Based Explainable Analyses

Samples from GSE122063 were analyzed using the R packages caret (7.0.1), Random Forest (4.7.1.2), kernlab (0.9.33), XGBoost (3.1.2.1), and klaR (1.7.3). A random seed of 12,345 was set, and the dataset was split into training and test sets at a ratio of 7:3. Five models (RF, SVM, XGBoost, GBM, and KNN) were trained using five-fold cross-validation. Model performance was evaluated by AUC and ROC curves with 95% confidence intervals were calculated using the pROC package (1.19.0.1). The model with the highest AUC was selected for subsequent SHAP analyses. SHAP values were calculated and visualized using the kernelshap (0.9.1) and shapviz packages (0.10.3) to assess the contribution of each gene to model prediction. Additional feature interpretation was performed using the DALEX package (2.5.3).

### 3.8. GO and KEGG Enrichment Analyses

Functional annotation of core genes was performed by Gene Ontology (GO) and Kyoto Encyclopedia of Genes and Genomes (KEGG) enrichment analyses using the clusterProfiler package (4.16.0) in R. An adjusted *p* < 0.05 was considered statistically significant. GO enrichment was evaluated across the Biological Process (BP), Cellular Component (CC), and Molecular Function (MF) categories. KEGG pathways were visualized using ggplot2 (4.0.0).

### 3.9. Mendelian Randomization Analyses

Mendelian randomization (MR) analyses were performed to evaluate the potential causal relevance of candidate genes in AD. Cis-expression quantitative trait loci (cis-eQTL) data were obtained from the eQTLGen consortium, which includes whole-blood samples from 31,684 individuals of European ancestry. AD GWAS summary statistics (ebi-a-GCST90027158), including 25,392 cases and 276,086 controls, were used as the outcome dataset. The R packages VariantAnnotation (1.54.1), gwasglue (0.0.0.9000), TwoSampleMR (0.6.19), qqman (0.1.9), and RadialMR (1.2.1) were used for the analyses. Exposure and outcome data were harmonized using the harmonise_data function. MR analyses were conducted using the mr function, and heterogeneity and pleiotropy were also assessed. Radial MR analyses were performed using the inverse-variance weighted (IVW) method.

### 3.10. Assessment of Differential Expression and Diagnostic Performance in External Datasets

To evaluate the performance of core genes in the external validation cohorts, receiver operating characteristic (ROC) curve analyses were performed and AUC values were calculated. Boxplots were also generated to visualize expression differences in core genes between groups in the validation datasets.

### 3.11. Immune Infiltration Analyses

Immune cell infiltration in AD and control groups was quantified using single-sample gene set enrichment analysis (ssGSEA) implemented in the GSVA package (v1.46.0). Between-group differences were assessed using the Wilcoxon rank-sum test. Associations between core gene expression and immune infiltration scores were further examined.

### 3.12. Single-Cell RNA-Seq Analyses

The single-cell RNA-seq dataset GSE175814 was downloaded from GEO and analyzed using Seurat (version ≥ 4.0). Quality control criteria included genes expressed in more than 200 cells and mitochondrial gene percentage < 20%. Expression data were normalized using LogNormalize with a scale factor of 10,000, and the top 2500 highly variable genes were identified using the VST method. Based on PCA results, KNN and Louvain clustering were performed using FindNeighbors and FindClusters (resolution = 0.3). Visualization was conducted by UMAP and PCA. Cell-type annotation was performed based on marker genes from the Cell Annotation file, followed by manual verification. Marker genes for each cluster were identified using FindAllMarkers (logFC > 1, adjusted *p* < 0.05, min.pct = 0.2). Pseudotime analysis was performed using monocle3. Cell–cell communication analysis was conducted using CellChat based on CellChatDB.human, retaining only cell types with at least 10 cells. All analyses were performed in R 4.5.1, and *p* < 0.05 was considered statistically significant.

### 3.13. Spatial Transcriptomic Analyses

Spatial transcriptomic data from GSE220442 were analyzed using Seurat (v4.0). Data normalization was performed with SCTransform, followed by PCA with 30 principal components retained. Clustering was carried out using FindNeighbors and FindClusters (resolution = 0.6). Marker genes were identified using FindAllMarkers (|logFC| > 1, adjusted *p* < 0.05, min.pct = 10%). Cell types were manually annotated based on enrichment scores from a reference database. Developmental trajectories were inferred using Monocle3 after conversion of the Seurat object to a CDS object. Differential expression analyses were performed using FindMarkers (4.2.0), and spatially variable genes were identified using Moran’s I. Visualization was generated using ggplot2 and ComplexHeatmap (2.25.1).

### 3.14. Molecular Docking

The three-dimensional structure of HSD17B1 (PDB ID: 6MNE) was retrieved from the Protein Data Bank, and the molecular structure of nobiletin was obtained from PubChem. The structures were converted into Mol2 format using Open BabelGUI (3.1.1). After protein preprocessing, including dehydration and hydrogenation, docking simulations were performed using AutoDockTools-1.5.7 and AutoDock Vina (1.5.7). The resulting complexes were converted into PDB format and visualized using PyMOL (3.2). The conformation with the highest predicted binding affinity was selected for further analysis.

### 3.15. Molecular Dynamics Simulation

A 100 ns molecular dynamics (MD) simulation was performed using Gromacs 2022 with the CHARMM36 force field. The protein–ligand complex was placed in a cubic simulation box with periodic boundary conditions and solvated using the TIP3P water model with a boundary distance of 1.0 nm. Electrostatic interactions were treated using the particle mesh Ewald (PME) method. The system was equilibrated through NVT and NPT ensembles before the production run. The final production simulation was performed at 310 K and 1 bar.

### 3.16. Cell Culture and Experimental Design

Human neuroblastoma SH-SY5Y cells were obtained from a certified cell bank and routinely verified to be free of mycoplasma contamination. Cells were cultured in DMEM/F-12 supplemented with 10% fetal bovine serum and 1% penicillin–streptomycin at 37 °C in 5% CO_2_. To establish an AD-like model, Aβ_1–42_ oligomers were prepared using a standard oligomerization protocol. SH-SY5Y cells were treated with Aβ_1–42_ oligomers (2 μM) for 24 h. Nobiletin (purity ≥ 98%) was dissolved in DMSO and used at a final concentration of 20 μM. Cells were divided into three groups: Control, AD, and AD + NOB. In the AD + NOB group, cells were pretreated with nobiletin for 2 h and then co-treated with nobiletin and Aβ_1–42_ for 24 h. At the end of treatment, cells were harvested for Western blot and qRT-PCR analyses. All experiments were performed with at least three independent biological replicates.

### 3.17. Western Blot Analysis

Following treatment, SH-SY5Y cells were lysed in RIPA buffer containing protease and phosphatase inhibitors. Protein lysates were collected after centrifugation, and protein concentration was measured using a BCA assay. Equal amounts of protein (20–30 μg per lane) were separated by 12% SDS–PAGE and transferred to PVDF membranes. Membranes were blocked with 5% non-fat milk in TBST and incubated overnight at 4 °C with primary antibodies against HSD17B1 (1:1000) and β-actin (1:5000). After incubation with HRP-conjugated secondary antibodies, immunoreactive bands were visualized using enhanced chemiluminescence and quantified with ImageJ (1.54r). HSD17B1 expression was normalized to β-actin.

### 3.18. RNA Extraction and Quantitative Real-Time PCR

Total RNA was extracted using TRIzol reagent (Invitrogen, Carlsbad, CA, USA) and quantified by spectrophotometry. Reverse transcription was performed using a kit with genomic DNA removal. Quantitative real-time PCR was carried out using a SYBR Green-based master mix. Each biological replicate was measured in technical triplicate, and no-template and no-reverse-transcription controls were included. Primer sequences were as follows: HSD17B1-F, GCTGCTACTTCCTGCTGATG; HSD17B1-R, CAGGATGATGGTGAGGATGG; ACTB-F, CACCATTGGCAATGAGCGGTTC; and ACTB-R, AGGTCTTTGCGGATGTCCACGT. Relative mRNA expression was calculated using the 2^−^ΔΔCt method with ACTB as the internal control. Data are presented as mean ± SD from at least three independent biological replicates.

## 4. Conclusions

This study identified HSD17B1 as a prioritized AD-associated candidate and a plausible nobiletin-responsive molecular node through an integrated multi-layered framework. By combining transcriptomic analyses, machine learning, Mendelian randomization, immune infiltration analyses, single-cell and spatial transcriptomics, structural modeling, and experimental validation, our findings support the relevance of HSD17B1 in AD and suggest a potential connection between nobiletin and neurosteroid-related regulation.

Overall, these findings provide a mechanism-oriented basis for further investigation of HSD17B1 in AD and support an integrated strategy for target prioritization in neurodegenerative disease research.

## Data Availability

Publicly available datasets were analyzed in this study. Bulk transcriptomic data were obtained from the Gene Expression Omnibus (GEO) under accession numbers GSE122063, GSE109887, and GSE132903. Single-cell RNA-seq data were obtained from GEO under GSE175814, and spatial transcriptomics data were obtained from GEO under GSE220442.

## Supplementary Materials

The following supporting information can be downloaded at: https://www.mdpi.com/article/10.3390/ijms27114756/s1.

## Abbreviations

The following abbreviations are used in this manuscript:

AD	Alzheimer’s disease
AI	Artificial intelligence
ARIA	Amyloid-related imaging abnormalities
AUC	Area under the curve
BBB	Blood–brain barrier
BP	Biological process
CC	Cellular component
CI	Confidence interval
CNS	Central nervous system
CTD	Comparative Toxicogenomics Database
DEGs	Differentially expressed genes
E1	Estrone
E2	Estradiol
eQTL	Expression quantitative trait loci
GBM	Gradient boosting machine
GEO	Gene Expression Omnibus
GO	Gene Ontology
GWAS	Genome-wide association study
HRT	Hormone replacement therapy
HSD17B1	Hydroxysteroid 17-β dehydrogenase 1
ICE	Individual conditional expectation
IVW	Inverse variance weighted
KEGG	Kyoto Encyclopedia of Genes and Genomes
Lasso	Least absolute shrinkage and selection operator
MD	Molecular dynamics
MF	Molecular function
MR	Mendelian randomization
NK cells	Natural killer cells
PCA	Principal component analysis
PDB	Protein Data Bank
PPI	Protein–protein interaction
RF	Random Forest
Rg	Radius of gyration
RMSD	Root mean square deviation
RMSF	Root mean square fluctuation
ROC	Receiver operating characteristic
SASA	Solvent accessible surface area
scRNA-seq	Single-cell RNA sequencing
SHAP	SHapley Additive exPlanations
SMILES	Simplified molecular input line entry system
SNP	Single nucleotide polymorphism
ssGSEA	Single-sample gene set enrichment analysis
SVM	Support vector machines
TOM	Topological overlap matrix
UMAP	Uniform manifold approximation and projection
WGCNA	Weighted gene co-expression network analysis
NOB	Nobiletin
