# Supplementary material for: Multi-Omics Integration and Causal Inference Identify HSD17B1 as a Potential Nobiletin Target Linking Neurosteroid Metabolism to Alzheimer’s Disease
Source: Int J Mol Sci. 2026 May 25;27(11):4756. doi: 10.3390/ijms27114756 (PMC13256803; doi:10.3390/ijms27114756)
Supplement: Supplementary file 1 [file ijms-27-04756-s001.zip › ijms-4243302-supplementary.pdf]

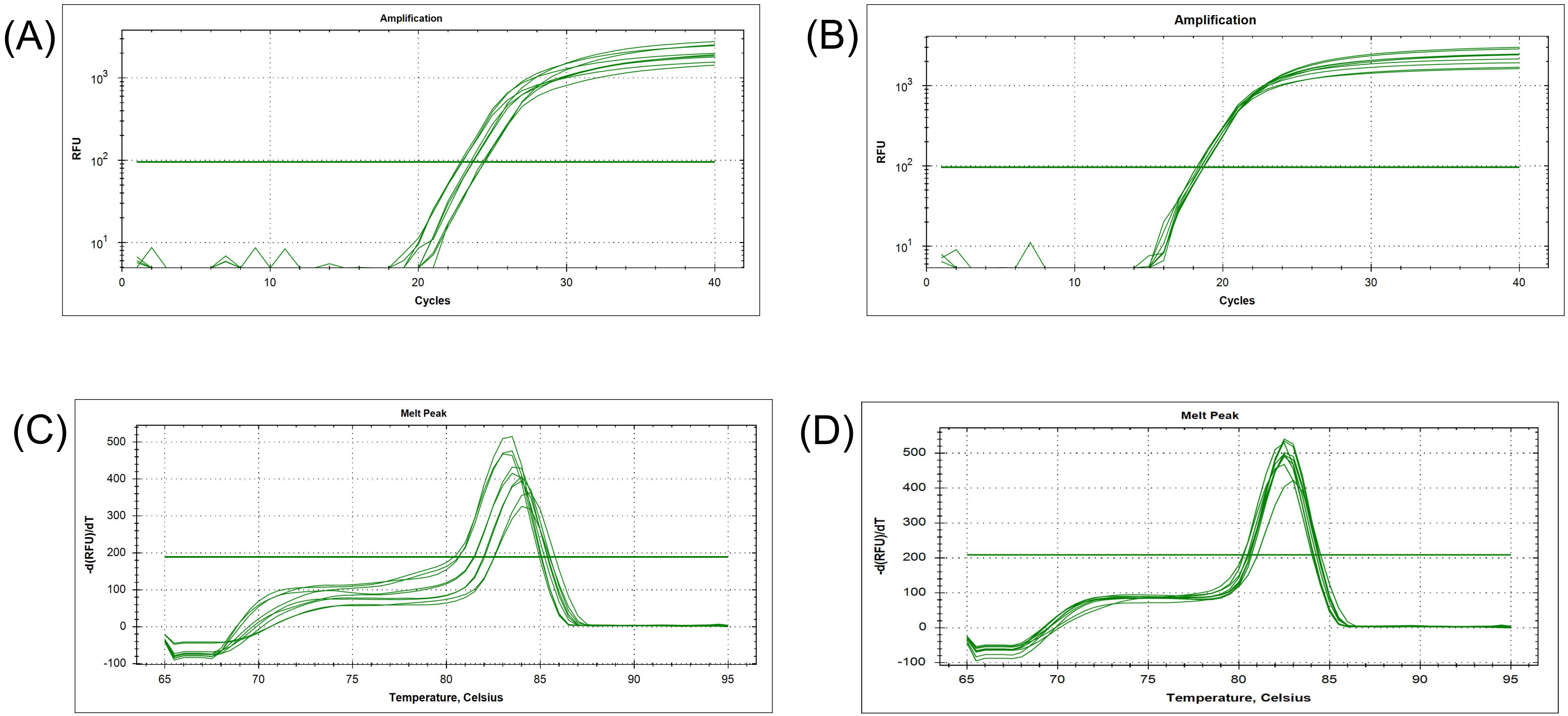

Figure S1. (**A**) Amplification Plot HSD17B1, (**B**) Amplification Plot β-actin, (**C**) Melt Curve Plot HSD17B1, (**D**) Melt Curve Plot β-actin.

(A)

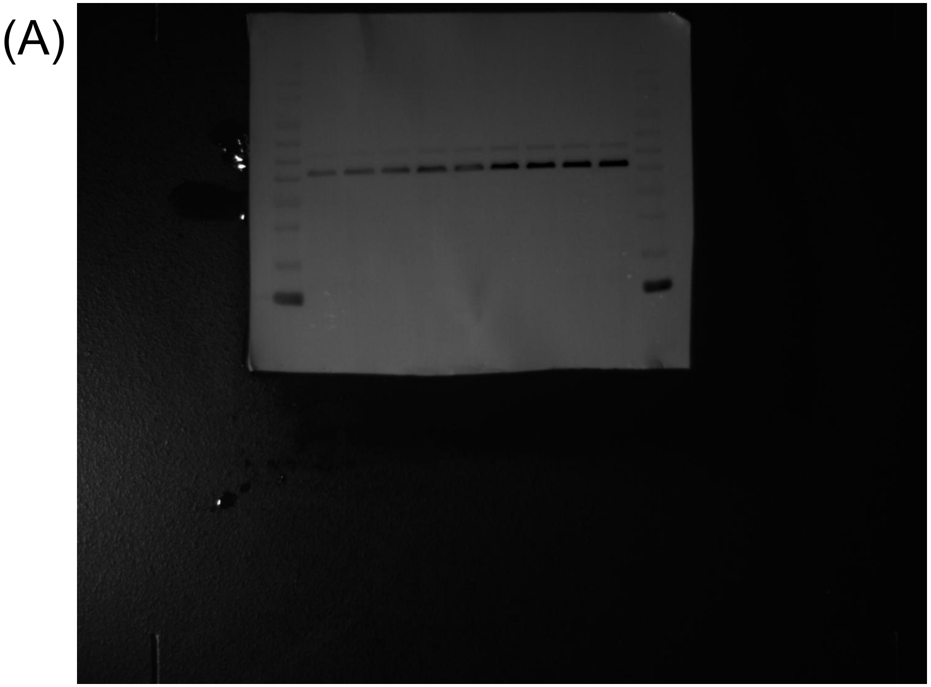

(B)

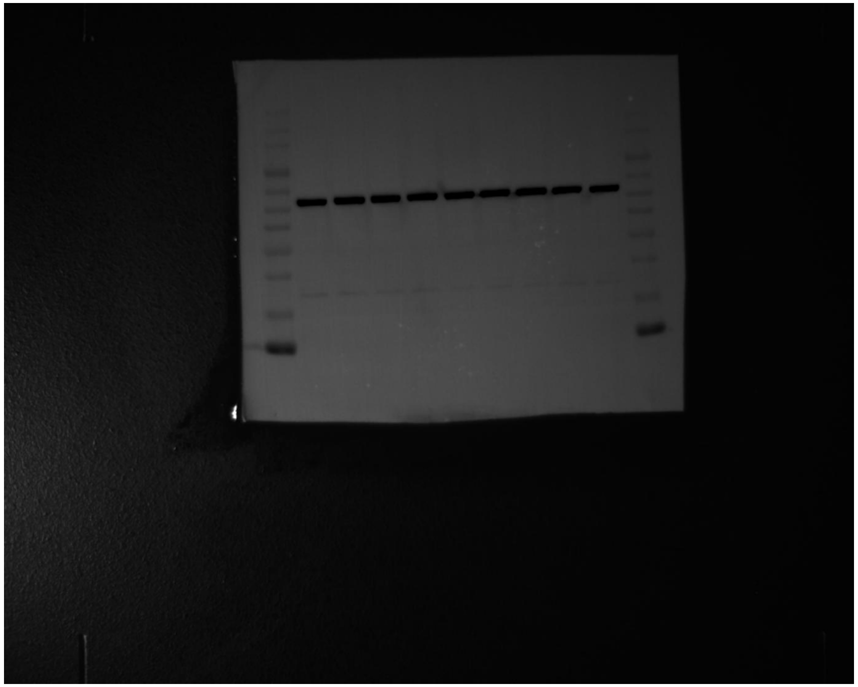

Figure S2. (**A**) original full-length blot HSD17B1, (**B**) original full-length blot β-actin.
